# Current status and trend in training for endoscopic submucosal dissection: A nationwide survey in Korea

**DOI:** 10.1371/journal.pone.0232691

**Published:** 2020-05-08

**Authors:** Jae Gon Lee, Chan Hyuk Park, Hyunsoo Chung, Jun Chul Park, Do Hoon Kim, Bo-In Lee, Jeong-Sik Byeon, Hwoon-Yong Jung

**Affiliations:** 1 Department of Internal Medicine, Hanyang University Guri Hospital, Hanyang University College of Medicine, Guri, Korea; 2 Division of Gastroenterology, Department of Internal Medicine and Liver Research Institute, Seoul National University College of Medicine, Seoul, Korea; 3 Division of Gastroenterology, Department of Internal Medicine, Severance Hospital, Yonsei University College of Medicine, Seoul, Korea; 4 Department of Gastroenterology, University of Ulsan College of Medicine, Asan Medical Center, Seoul, Korea; 5 Division of Gastroenterology, Department of Internal Medicine, College of Medicine, The Catholic University of Korea, Seoul, Korea; Lundquist Institute at Harbor-UCLA Medical Center, UNITED STATES

## Abstract

**Background:**

Although endoscopic submucosal dissection (ESD) is widely used, the current status and trend in its training have yet to be fully evaluated. We aimed to investigate how ESD endoscopists have been trained in actual clinical practice.

**Methods:**

Endoscopists aged <45 years who have completed a gastroenterology fellowship or were currently in a fellowship for ≥2 years were included. We conducted a nationwide survey on the ESD training experiences of these endoscopists.

**Results:**

Among 79 young Korean endoscopists invited to participate in the survey, 68 (86.1%) trained in 24 major hospitals responded to the questionnaire. Twenty, 25, and 23 participants belonged to the second-year fellow, <5 years after training, and ≥5 years after training groups, respectively. Sixty-nine percent of the participants observed ≥50 ESD cases before starting ESD under supervision by an expert endoscopist. Additionally, 22% experienced ≥20 supervised ESDs during the training period. The proportion of the participants who underwent a hands-on course differed among the groups (≥5 years after training, 13.0%; <5 years after training, 40.0%; and second-year fellow, 50.0%; *P* = 0.027). ESD under supervision, observation, and hands-on course were the preferred methods for learning ESD (91.1%, 80.9%, and 35.3%, respectively). Overall, 42.6% of the participants were satisfied with their training program. More experience in supervised ESD (≥20 cases) was associated with an increased satisfaction (odds ratio, 6.65; 95% confidence interval, 1.62–36.31).

**Conclusions:**

Observation and performance of ESD under the supervision of an expert endoscopist are the primary methods for learning ESD. Hands-on course program has been used more frequently in recent years.

## Introduction

Endoscopic submucosal dissection (ESD) is currently widely used as a minimally invasive resection technique for superficial gastrointestinal neoplasms [1]. Recently, detection of early gastrointestinal neoplasms is increasing with the widespread use of screening gastrointestinal endoscopy; the implementation of ESD is also increasing accordingly [2,3]. According to previous studies using a national database on ESD in Japan, approximately 13,000 gastric ESD, 2,000 esophageal ESD, and 8,000 colorectal ESD procedures were performed annually [4–6]. ESD in Western countries is not as widely practiced as that in East Asian countries; however, its implementation is increasing in recent years [7].

ESD is advantageous as compared with laparotomy or laparoscopic surgery in that it does not require general anesthesia, has a rapid recovery after the procedure, and shortens the length of hospital stay [8]. However, there are risks of procedure-related complications and incomplete resection, especially when performed by a less experienced practitioner [9]. Therefore, it is generally accepted that ESD should be performed by a properly trained endoscopist [1].

Although there have been several reports on ESD training programs, each study has been conducted with individual institutions’ own training programs [10–12]. In addition, there are differences in the operating conditions of ESD among countries because of the differences in the prevalence of gastrointestinal cancers; availability of healthcare resources, including endoscopic devices; and socioeconomic status [7,13]. To perform safe procedures and obtain optimal outcomes even in countries where ESD is newly introduced or not widely available, a consensus on ESD training programs needs to be drawn.

Prior to this, it is important to know how ESD endoscopists have been trained in actual clinical practice. In particular, investigating the current status of ESD training in Korea can help build a proper training environment for many countries that want to train ESD experts in the future. In Korea, ESD has been actively implemented since 2003 and is currently performed in many institutions, including 45 tertiary hospitals [3]. Endoscopists who have been trained in the early period of ESD introduction currently offer ESD training to novice endoscopists, and a systematic and efficient ESD training program is being developed in each hospital. However, nationwide data on the current status of ESD training programs are lacking. Therefore, we conducted a nationwide survey for young endoscopists in Korea regarding their ESD training experiences.

## Methods

### Study design

We retrospectively analyzed the previously performed survey results for evaluating current status of ESD training program in Korea. Gastrointestinal endoscopists from 31 university-affiliated hospitals and tertiary care medical centers in Korea were invited to participate in the survey. Endoscopists aged <45 years who have completed a gastroenterology fellowship training or who were currently in a fellowship training for ≥2 years were included. The survey was conducted online using Google Forms (https://docs.google.com/forms) for 2 weeks in December 2018 (**S1 Appendix**).

### Survey questionnaire

The questionnaire consisted of 42 questions. The baseline characteristics of the participants, including demographic information, duration of fellowship training, current clinical position, major fields of clinical practice in gastroenterology, and major fields of ESD, were investigated. The items on the training methods included the timing and number of observations for ESD before starting ESD under supervision and the timing and number of ESD implementation under supervision. The preferred resources/methods for learning ESD, including observation, symposium or conference, live demonstration, literature, video, hands-on course, and experience in ESD under supervision of an expert endoscopist, were also investigated. Additionally, we surveyed opinions on the number of observations and ESD implementation under supervision required for achieving competency in independent ESD.

The satisfaction with the overall training program or experience of a hands-on course was assessed using a five-point Likert scale. Four or five points in the five-point Likert scale was considered to indicate satisfaction.

### Statistical analysis

Continuous variables were expressed as means ± standard deviations and categorical variables as numbers with proportions. To evaluate the trend in the training methods and trainees’ awareness, we classified the participants into three groups as follows: (a) second-year fellow, (b) <5 years after fellowship training, and (c) ≥5 years after fellowship training. The continuous and categorical variables among the groups were compared using the analysis of variance and chi-square or Fisher’s exact test, respectively.

To identify the factors associated with satisfaction with ESD training, a logistic regression analysis was performed. Significant factors in the univariable analysis and potential confounding variables, including age, sex, timing and number of ESD observations, and experience of a hands-on course, were adjusted in the multivariable analysis. A *P* value of <0.05 was considered statistically significant. All statistical procedures were conducted using R (version 3.5.2; R Foundation for Statistical Computing, Vienna, Austria).

### Ethics statement

We informed the participants that the survey will be conducted anonymously, and any personal information will not be disclosed publicly. All participants provided written informed consent. The Institutional Review Board on Human Subjects Research and Ethics Committee, Hanyang University Guri Hospital waived review of the current study protocol because the survey had been already performed anonymously before starting retrospective analysis in this study (GURI 2019-04-019).

## Results

### Baseline characteristics of the participants

Among 79 young endoscopists from 31 hospitals in Korea invited to participate in the survey, 68 (86.1%) responded to the questionnaire. The baseline characteristics of the participants are shown in **Table 1**. Their mean age was 37.2 years, and 67.6% were men. The participants had been trained previously or are currently being trained in 24 hospitals in Korea. Twenty (29.4%), 25 (36.8%), and 23 (33.8%) participants were classified into the second-year fellow, <5 years after fellowship training, and ≥5 years after fellowship training groups, respectively. The majority of the participants responded that they are currently practicing in the field of the upper (73.5%) or lower (63.2%) gastrointestinal tract, and the major field of ESD was the stomach (83.8%). **S1 Table** shows differential characteristics according to the current position.

**Table 1:** Baseline characteristics of the included participants.

Variable			Value
Number, n			68
Age, year, mean±SD			37.2±3.4
Sex, n (%)			
	Male		46 (67.6)
	Female		22 (32.4)
Number of training hospital, n			24
Current position, n (%)			
	Second-year fellow		20 (29.4)
	Faculty		48 (70.6)
		< 5 years after fellowship training	25 (36.8)
		≥ 5 years after fellowship training	23 (33.8)
Duration of fellowship training, n (%)^a^			
	1 year		5 (10.4)
	2 years		34 (70.8)
	3 years		5 (10.4)
	4 years		2 (4.2)
	5 years		2 (4.2)
Major field of clinical practice, n (%)^b^			
	Upper GI tract		50 (73.5)
	Lower GI tract		43 (63.2)
	Pancreatobiliary system		5 (7.4)
	Liver		1 (1.5)
	GI motility disease		4 (5.9)
Major field of ESD, n (%)^b^			
	Esophagus		19 (27.9)
	Stomach		57 (83.8)
	Duodenum		4 (5.9)
	Colon		26 (38.2)
	Rectum		30 (44.1)

^a^Only endoscopists who have completed fellowship training were included in this variable.

^b^Multiple responses were permitted.

GI, gastrointestinal; ESD, endoscopic submucosal dissection; SD, standard deviation

### Training experience in the hospital

**Table 2** shows the participants’ experience in ESD training in the hospital. The participants in the ≥5 years after fellowship training group completed their fellowship training between 2007 and 2012. The participants in the <5 years after fellowship training group finished their fellowship training between 2012 and 2016. The participants in the second-year fellow group are currently being trained since 2017.

**Table 2:** Experience in endoscopic submucosal dissection training in the hospital.

Variable			Second-year fellow	< 5 years after fellowship training	≥ 5 years after fellowship training	*P*-value
Number, n			20	25	23	
Year of training period			2017–2018	2012–2016	2007–2012	
Observation, n (%)						
	First observation of ESD					0.005
		At the training of residency	4 (20.0)	10 (40.0)	7 (30.4)	
		At the first-year fellow	15 (75.0)	12 (48.0)	5 (21.7)	
		At the second-year fellow	1 (5.0)	3 (12.0)	6 (26.1)	
		At the third-year (or higher) fellow	N/A	0 (0.0)	4 (17.4)	
		After the training of fellow	N/A	0 (0.0)	1 (4.3)	
	Additional role during observation					0.033
		Observation only	1 (5.0)	7 (28.0)	9 (39.1)	
		ESD assistance with observation^a^	19 (95.0)	18 (72.0)	14 (60.9)	
	Total number of observation before starting ESD under supervision					0.124
		0–9	0 (0.0)	0 (0.0)	4 (17.4)	
		10–49	6 (30.0)	6 (24.0)	5 (21.7)	
		50–99	7 (35.0)	4 (16.0)	4 (17.4)	
		≥100	7 (35.0)	15 (60.0)	10 (43.5)	
ESD under supervision of an expert endoscopist, n (%)						
	First ESD under supervision					<0.001
		At the training of residency	0 (0.0)	1 (4.0)	0 (0.0)	
		At the first-year fellow	3 (15.0)	0 (0.0)	1 (4.3)	
		At the second-year fellow	13 (65.0)	18 (72.0)	10 (43.5)	
		At the third-year (or higher) fellow	0 (0.0)	4 (16.0)	2 (8.7)	
		After the training of fellow	0 (0.0)	2 (8.0)	8 (34.8)	
		No experience of ESD under supervision	4 (20.0)	0 (0.0)	2 (8.7)	
	Total number of ESD under supervision					0.007
		0	4 (20.0)	0 (0.0)	2 (8.7)	
		1–9	12 (60.0)	8 (32.0)	13 (56.5)	
		10–19	2 (10.0)	10 (40.0)	2 (8.7)	
		20–29	2 (10.0)	3 (12.0)	1 (4.3)	
		≥30	0 (0.0)	4 (16.0)	5 (21.7)	
	Tumor location in ESD under supervision^b^					
		Esophagus	0 (0.0)	1 (4.0)	1 (4.3)	>0.999
		Upper third of the stomach	0 (0.0)	5 (20.0)	6 (26.1)	0.035
		Middle third of the stomach	2 (10.0)	11 (44.0)	8 (34.8)	0.044
		Lower third of the stomach	16 (80.0)	23 (92.0)	15 (65.2)	0.072
		Duodenum	0 (0.0)	1 (4.0)	1 (4.3)	>0.999
		Colon	0 (0.0)	3 (12.0)	1 (4.3)	0.378
		Rectum	0 (0.0)	5 (20.0)	3 (13.0)	0.105

^a^Assistance includes patient monitoring, administration of sedative agent, and control of accessory device such as injection needle.

^b^Multiple responses were permitted.

ESD, endoscopic submucosal dissection; N/A, not applicable

Most participants started ESD observation from their first-year fellowship or residency. Especially, 95.0% and 88.0% of the participants in the second-year fellow group and <5 years after fellowship training group, respectively, experienced their first ESD observation at their first-year fellowship or residency. However, less participants in the ≥5 years after fellowship training group started ESD observation at their first-year fellowship or residency compared with the other groups (*P* = 0.005).

In the second-year fellow group, 95% of the participants served as assistants during ESD observations. In contrast, in the <5 years after fellowship training and ≥5 years after fellowship training groups, 72.0% and 60.9% of the participants served as assistants (*P* = 0.033). The majority of the participants responded that they have observed ≥50 procedures; there was no difference among the groups.

In the second-year fellow and <5 years after fellowship training groups, the majority of the participants experienced their first ESD procedure under supervision by an expert endoscopist during their fellowship (second-year fellow, 80%; <5 years after fellowship training, 88%). On the contrary, 56.5% of the participants in the ≥5 years after fellowship training group performed their first supervised ESD during their fellowship, and 34.8% experienced their first supervised ESD after finishing their fellowship (*P*<0.001).

Regarding the total number of ESDs performed under supervision, the participants in the <5 years after fellowship training group experienced more procedures than did those in the second-year fellow group. Eighty percent of the second-year fellow group participants experienced <10 procedures, whereas 68% of the <5 years after fellowship training group participants experienced ≥10 procedures. Meanwhile, in the ≥5 years after fellowship training group, experience in ESD under supervision varied: 65.2% experienced <10 procedures, while 21.7% experienced ≥30 procedures.

Eighty percent of the participants in the second-year fellow group experienced gastric ESD during the training period; however, no participant experienced ESD of the esophagus, upper third of the stomach, and colorectum.

### Learning resources/methods for ESD training

Overall, 73.5% of the participants considered ESD under supervision as the most important learning method for ESD training (**Table 3**). When asked to respond to a series of three preferred learning methods for ESD training, ESD under supervision, observation, and hands-on course were highly rated (91.1%, 80.9%, and 35.3%, respectively).

**Table 3:** Experience of learning resources for endoscopic submucosal dissection training.

Variable		Second-year fellow	< 5 years after fellowship training	≥ 5 years after fellowship training	*P*-value
Number, n		20	25	23	
The most wanted method for learning ESD, n (%)					0.512
	Observation of ESD	2 (10.0)	5 (20.0)	5 (21.7)	
	Symposium or conference	0 (0.0)	0 (0.0)	1 (4.3)	
	Live demonstration	0 (0.0)	0 (0.0)	1 (4.3)	
	Literature (*e*.*g*., journal, book)	0 (0.0)	1 (4.0)	1 (4.3)	
	Video (*e*.*g*., YouTube)	0 (0.0)	0 (0.0)	0 (0.0)	
	Hands-on course	2 (10.0)	0 (0.0)	1 (4.3)	
	ESD under supervision of an expert endoscopist	16 (80.0)	19 (76.0)	15 (65.2)	
Preferred methods for learning ESD, n (%)^a^					
	Observation of ESD	15 (75.0)	23 (92.0)	17 (73.9)	0.194
	Symposium or conference	2 (10.0)	3 (12.0)	4 (17.4)	0.819
	Live demonstration	5 (25.0)	5 (20.0)	8 (34.8)	0.503
	Literature (*e*.*g*., journal, book)	1 (5.0)	9 (36.0)	4 (17.4)	0.038
	Video (*e*.*g*., YouTube)	1 (5.0)	0 (0.0)	5 (21.7)	0.016
	Hands-on course	9 (45.0)	8 (32.0)	7 (30.4)	0.554
	ESD under supervision of an expert endoscopist	18 (90.0)	24 (96.0)	20 (87.0)	0.573
Methods that participants experienced for learning ESD^b^					
	Observation of ESD	20 (100.0)	25 (100.0)	23 (100.0)	N/A
	Symposium or conference	14 (70.0)	21 (84.0)	15 (65.2)	0.308
	Live demonstration	13 (65.0)	15 (60.0)	12 (52.2)	0.688
	Literature (*e*.*g*., journal, book)	14 (70.0)	22 (88.0)	14 (60.9)	0.095
	Video (*e*.*g*., YouTube)	5 (25.0)	12 (48.0)	9 (39.1)	0.286
	Hands-on course	10 (50.0)	10 (40.0)	3 (13.0)	0.027
	ESD under supervision of an expert endoscopist	16 (80.0)	25 (100.0)	21 (91.3)	0.056

^a^Each participants chose up to 3 items in this variable.

^b^Multiple responses were permitted.

ESD, endoscopic submucosal dissection; N/A, not applicable

During the training period, most participants experienced various learning resources, including observation, symposium or conference, live demonstration, and video. In most types of learning resources, there was no significant difference in the experience among the groups; however, experience of a hands-on course during the training period differed among the groups (second-year fellow, 50.0%; <5 years after fellowship training, 40.0%; and ≥5 years after fellowship training, 13.0%, *P* = 0.027).

Detailed data about experience and demand of hands-on course are shown in **S2 Table**. Training hospitals provided most of the hands-on course programs. However, participants also experienced hands-on model through the program by conference or endoscopic device company. The most frequently experienced hands-on model was the gastric ESD model. On the contrary, most participants wanted to experience a colorectal ESD model for ESD training. In the questionnaire item asking whether the participants had an experience of a hands-on course during or after the training period, 60.3% answered that they had such experiences (**S1 Fig**). More than 60% of the participants responded that the hands-on course helps beginners to improve their skills. Especially, 75% of the participants in the second-year fellow group agreed that the hands-on course is helpful to beginners for learning ESD.

### Satisfaction with training

**Fig 1:**
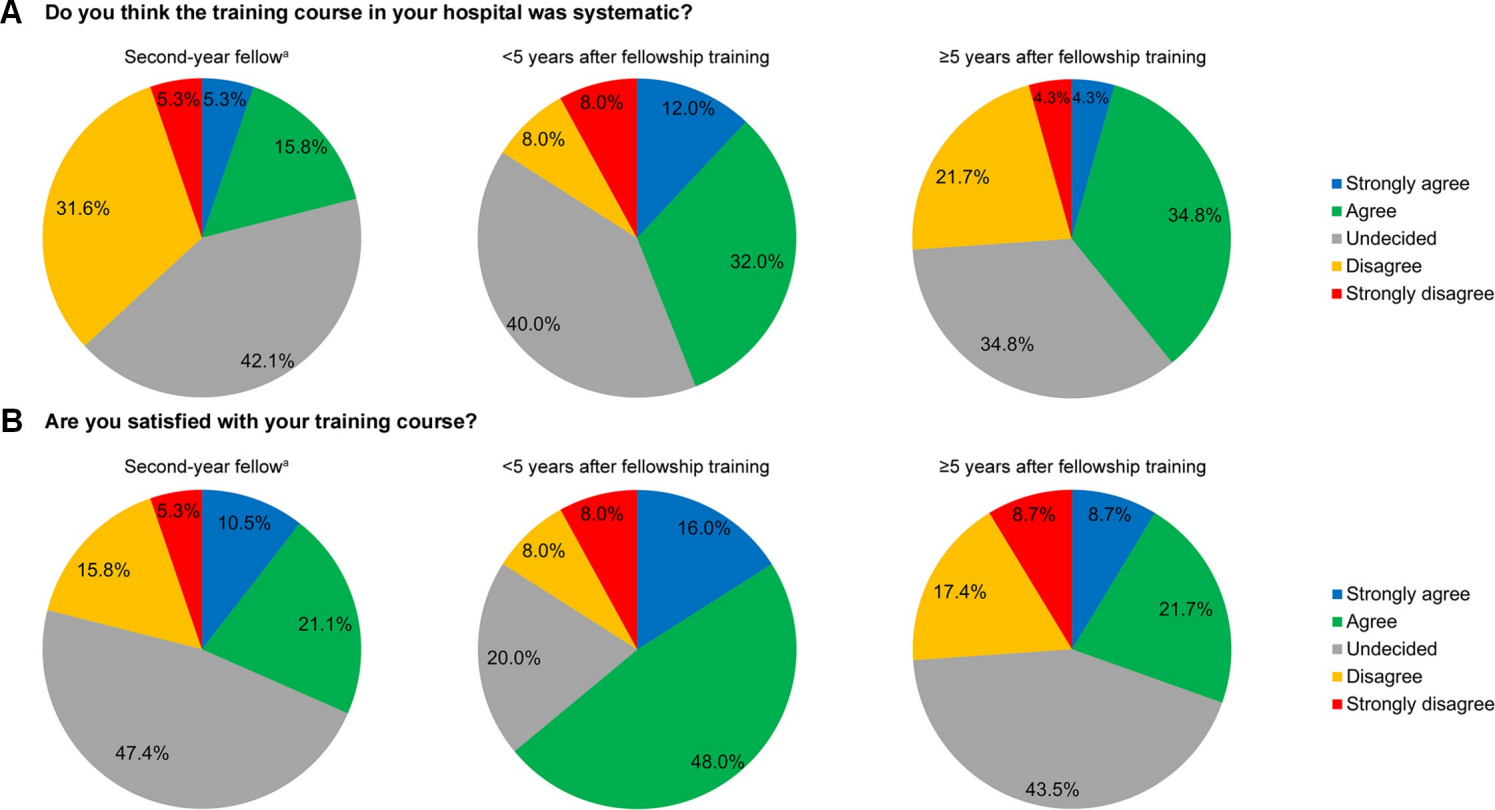
Satisfaction score for the training course in the hospital. (A) Score for the systematic training. The mean scores were 2.8, 3.3, and 3.1 in the second-year fellow group, <5 years after fellowship training group, and ≥5 years after fellowship training group, respectively (*P* = 0.401). (B) Score for the overall satisfaction. The mean scores were 3.2, 3.6, and 3.0 in the second-year fellow group, <5 years after fellowship training group, and ≥5 years after fellowship training group, respectively (*P* = 0.663). ^a^One participant in the second-year fellow group did not respond to these questions.

Only 35.3% of the total participants agreed (or strongly agreed) that the training program of their hospital was systematic (five-point Likert scale: mean, 3.1; standard deviation, 1.0). In addition, 42.6% of the participants were satisfied with their training program (five-point Likert scale: mean, 3.3; standard deviation, 1.1). The degrees of satisfaction according to the groups are shown in **Fig 1**.

In the univariable logistic regression model, early experience of the first supervised ESD (odds ratio [OR], 3.49; 95% confidence interval [CI], 1.15‒12.14) and ≥20 cases of supervised ESD (OR, 8.24; 95% CI, 2.27‒39.80) were identified as factors associated with satisfaction (**Table 4**). After adjusting for potential confounding variables, including age, sex, timing and number of ESD observation, and experience of a hands-on course during the training period, only ≥20 cases of supervised ESD was associated with an increased satisfaction (OR, 6.65; 95% CI, 1.62‒36.31).

**Table 4:** Factors associated with overall satisfaction (satisfaction score, ≥4) with endoscopic submucosal dissection training^a^.

Variable			Overall satisfaction n (%)	N	Univariable analysis		Multivariable analysis	
					OR (95% CI)	*P*-value	OR (95% CI)	*P*-value
Age, /year			N/A	N/A	1.02 (0.89–1.19)	0.741	0.96 (0.77–1.20)	0.723
Sex								
	Male		23 (50.0)	46	2.50 (0.85–8.07)	0.105	2.89 (0.62–15.76)	0.193
	Female		6 (28.6)	21	1		1	
Observation of ESD								
	Starting ESD observation							
		Early (from either residency or the first-year of fellowship)	25 (48.1)	52	2.55 (0.76–10.14)	0.148	2.08 (0.47–10.81)	0.350
		Late (from second-year of fellowship or later)	4 (26.7)	15	1		1	
	Number of ESD observation before staring ESD under supervision							
		<100 cases	16 (45.7)	35	1		1	
		≥100 cases	13 (40.6)	32	0.81 (0.30–2.14)	0.675	0.81 (0.24–2.69)	0.735
ESD under supervision								
	First case of ESD under supervision							
		Early (at the second-year of fellowship or earlier)	24 (52.2)	46	3.49 (1.15–12.14)	0.035	3.28 (0.84–14.71)	0.098
		Late (at the third-year of fellowship or later)	5 (23.8)	21	1		1	
	Number of ESD under supervision							
		<20 cases	17 (32.7)	52	1		1	
		≥20 cases	12 (80.0)	15	8.24 (2.27–39.80)	0.003	6.65 (1.62–36.31)	0.014
Experience of learning resources								
	Symposium or conference		23 (46.9)	49	1.77 (0.59–5.79)	0.322		
	Live demonstration		18 (46.2)	39	1.32 (0.50–3.61)	0.576		
	Literature (*e*.*g*., journal, book)		24 (49.0)	49	2.50 (0.81–8.77)	0.127		
	Video (*e*.*g*., YouTube)		13 (50.0)	26	1.56 (0.58–4.26)	0.378		
	Hands-on course		8 (36.4)	22	0.65 (0.22–1.84)	0.425	0.40 (0.10–1.42)	0.165

^a^One participant who did not respond to satisfaction score was excluded in this analysis.

ESD, endoscopic submucosal dissection; OR, odds ratio; CI, confidence interval; N/A, not applicable

Most participants who gave poor scores to questions regarding whether the training program was systematic or satisfactory responded that ESD training was provided depending on mentors, without a systematic program, for the subjective questions that asked for general opinions on their ESD training experience.

### Awareness of learning curves

**Fig 2:**
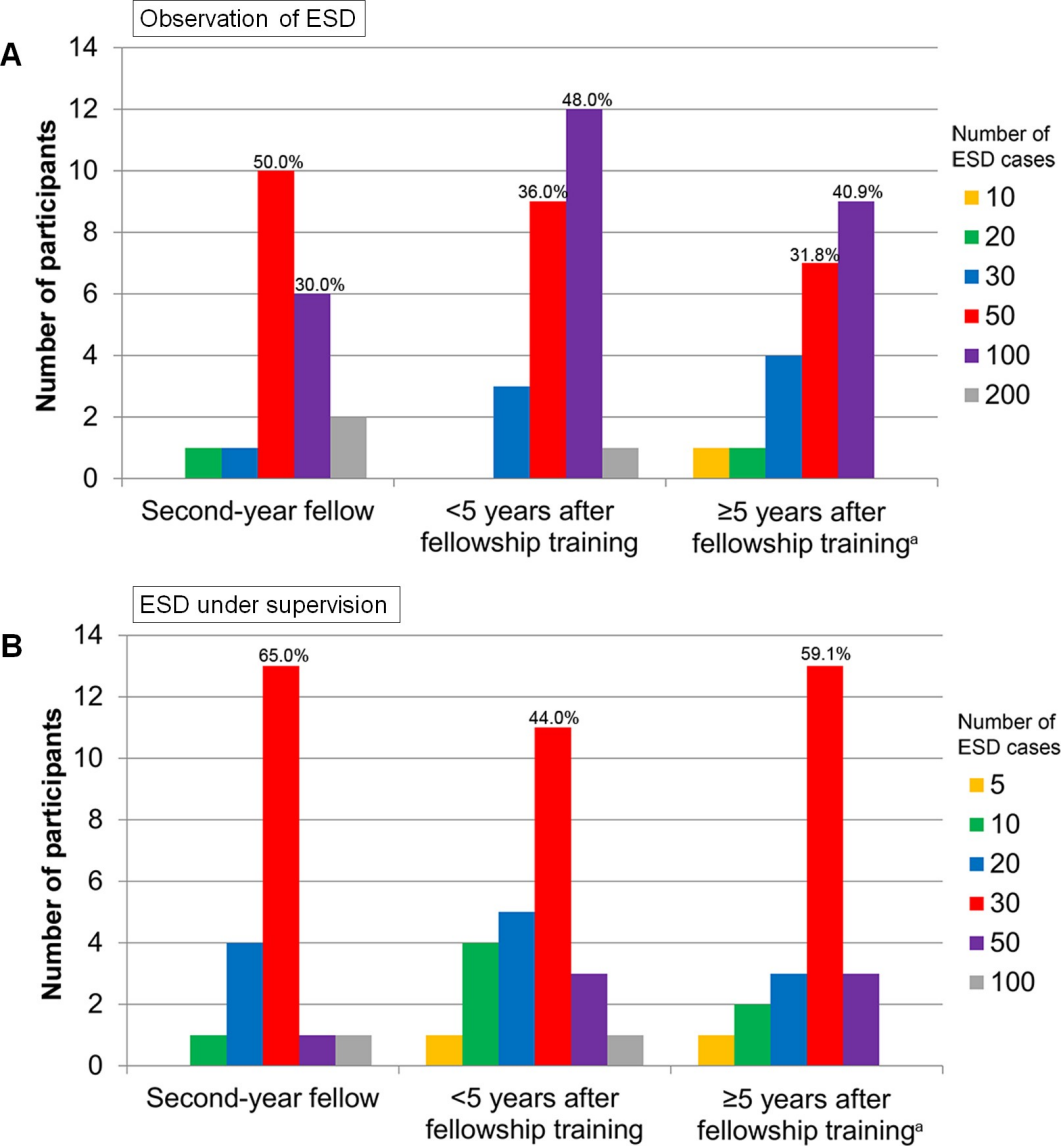
Optimal number of observations and procedures under supervision for learning ESD. (A) Optimal number of observations before starting ESD under supervision. (B) Optimal number of procedures under supervision to achieve competency. ^a^One participant in the ≥5 years after fellowship training group did not respond to these questions. ESD, endoscopic submucosal dissection.

The majority of the participants rated that the optimal number of observations before starting ESD under supervision is either 50 or 100 (**Fig 2**). Although it was not statistically significant, the second-year fellow group were likely to consider that the optimal number of observations is 50, whereas the <5 years and ≥5 years after fellowship training groups were more likely to consider 100 cases of observation are needed to start ESD under supervision.

The participants usually thought that the optimal number of supervised ESDs before the independent procedure is 30. Ten percent of the second-year fellow group participants and 14.9% of the <5 years and ≥5 years after fellowship training group participants responded that 50 or 100 supervised ESDs are needed; however, there was no significant difference.

## Discussion

In Korea, medical students complete a 4-year medical school course after finishing a 2-year premedical or a 4-year general undergraduate course. After graduating medical school, they perform a 1-year internship and 4-year residency program. After at least 1 or 2 years of fellowship training, they can become gastrointestinal endoscopy specialists. In this study, we investigated the current status and trend in ESD training in Korea using a nationwide survey. Most participants began observing ESD during their residency or first-year fellowship and have attended more than 50 procedures. The majority of the participants started the procedure under supervision at their second-year fellowship and experienced 10–20 cases. However, the overall satisfaction with the training was moderate, and less than half of the participants experienced a hands-on course in their training period.

In a previous study on an ESD training program, four ESD trainees performed 30 gastric ESDs under supervision for 2 years, with an *en bloc* resection rate of 100% and a self-completion rate of 80.3% [12]. In another study, trainees performed 30 gastric ESDs under supervision, with a completion rate of 93% and a complication rate of 4.4%, demonstrating that ESD can be safely performed by novice practitioners under the guidance of an expert [10]. In the present study, the majority of the participants experienced 10–20 cases of ESD under supervision, which are fewer than the suggested number of cases for achieving competency in previous studies. Less experience than trainees’ demand may have affected the moderate-degree satisfaction with the training program in our survey. The logistic regression analysis also showed that more experience in ESD under supervision was associated with the trainees’ satisfaction. However, we cannot conclude that the actual clinical practice in ESD training in Korea is absolutely incorrect. In a previous study, operators with <30 cases of ESD procedure showed a relatively long procedure time; however, the *en bloc* resection and complication rates were acceptable [11]. Competency may be achieved with relatively less experiences in ESD under supervision using various learning resources, especially hands-on courses.

Various gastrointestinal endoscopy training simulators have been developed [14]. Live porcine models are realistic compared to the human setting and highly regarded as a learning tool for esophageal and gastric ESD [15]. In our study, only 13% of the participants in the ≥5 years after fellowship training group, who had been trained about 5–10 years ago, experienced a hands-on course during their training period; conversely, 50% of the participants in the second-year fellow group experienced such. Previous studies on *ex vivo* porcine models for esophageal and colonic ESDs have suggested that 10 *ex vivo* procedures are required to achieve the learning curve [16,17]. *Ex vivo* hands-on models are preferred learning materials for ESD trainees because they are relatively easy to implement without imposing great pressure. Our survey also demonstrated that 75% of the trainees currently undergoing training (second-year fellow group) responded that the hands-on course would be helpful for ESD training. However, *ex vivo* models have limitations in experiencing peristalsis, intraluminal secretions, and bleeding during the procedure [18]. These disadvantages of *ex vivo* hands-on models can be complemented by subsequent *in vivo* animal model training [19].

Although all participants in this study experienced procedure observation, the experience of learning through literature, lectures at the symposium, or video was less common. Given that a stepwise training algorithm has suggested that acquisition of basic knowledge and skills in ESD is the first step of training [13], it may be inappropriate to learn ESD via observation alone. Although oral education for basic knowledge of the procedure takes place during observation in the field, the importance of systematic learning through literature and lectures cannot be overemphasized. Effective image training can be achieved with observations with theoretic background regarding the overall process, instruments, and management of complications.

When ESD was first introduced, there was no established protocol or experts in the field. Therefore, the early pioneers have built up their skills by themselves while practicing their own procedures. However, as there are already established experts in ESD, it is generally accepted that only endoscopists who have acquired their skills under the guidance of an expert and have minimal requirements should perform the procedure [20]. ESDs conducted by endoscopists who are not adequately trained may cause harm to the patients and may result in ethical problems [13]. Therefore, we encourage education and training through hands-on models before starting ESD.

One of the interesting findings of our study is that the only factor influencing the satisfaction with training was the experience in ESD under supervision. Hands-on courses and live demonstrations were analyzed to have no effect on satisfaction. Although hands-on courses may play an important role in the early phase of the training course, experience in ESD under supervision by an expert is ultimately necessary for successful and satisfactory training. However, this does not mean that apprenticeship education is sufficient for ESD training. Some trainees were dissatisfied with apprenticeship education alone. The introduction of a systematic training program is necessary to improve training program satisfaction.

In addition to the abovementioned points, differences in the incidence of early gastrointestinal neoplasms by organs (esophagus, stomach, or colorectum) can be an issue in ESD training. In Korea and Japan, early gastric neoplasms are prevalent; therefore, trainees can start ESD training with procedures targeting gastric adenoma or early gastric cancer in the antrum, which are relatively easy to perform and less likely to yield complications. However, the incidence of superficial gastric neoplasms in some countries is low; conversely, that of superficial esophageal or colorectal neoplasms is relatively high [21,22]. In these regions, ESD training may be started with rectal ESD of relatively small lesions.

Although our study provided data on ESD training in the actual clinical practice in Korea, it has several limitations. First, the survey was not a complete enumeration survey. Although we invited as many young endoscopists as possible from major general hospitals in Korea, some ESD endoscopists in clinics may not have been included in the survey. However, the response rate in our survey was relatively high, and the number of respondents was sufficient to analyze the status of ESD training in Korea. Second, recall bias may be a concern in our study because the study was not designed as a prospective study. The survey response of the endoscopists who completed the training several years ago (i.e., ≥5 years after fellowship training group) may be less accurate. Nevertheless, we believe that the results of our survey are reliable because the participants in the <5 years and ≥5 years after fellowship training groups are currently working in the major hospitals in Korea. They may be always interested in educating their trainees on ESD and remember their experience during the ESD training period. Additionally, the survey planning step did not consider the specific type of hands-on models. Therefore, we could not obtain information about the type of hands-on models used by participants. Future surveys may require more specific questionnaires.

Despite these limitations, our study provides a better understanding of the current status and trend in ESD training in Korea. Young endoscopists gain ESD skills using various learning resources. Among them, observation and performance of ESD under the supervision of an expert endoscopist are the primary methods for learning ESD. Training through a hands-on course has been used more frequently in recent years. The survey data in the current study will be an important basis for establishing or upgrading ESD training programs worldwide. Unified and structured ESD training programs should be established for the safe and effective endoscopic treatment of early gastrointestinal neoplasms.

## Supporting information

S1 Fig Experience of a hands-on course. (A) Overall experience of a hands-on course during or after the training period. (B) Awareness of a hands-on course. The mean scores were 4.1, 3.8, and 3.6 in the second-year fellow, <5 years after fellowship training, and ≥5 years after fellowship training groups, respectively (*P* = 0.067). ESD, endoscopic submucosal dissection.
(TIF)

S1 Table Differential characteristics according to the current position. (DOCX)

S2 Table Experience and demand of hands-on course. (DOCX)

S1 Appendix Survey questionnaire. (DOCX)
