# Supplementary figures and images for: Current status and trend in training for endoscopic submucosal dissection: A nationwide survey in Korea
Source: PLoS One. 2020 May 8;15(5):e0232691. doi: 10.1371/journal.pone.0232691 (PMC7209322; doi:10.1371/journal.pone.0232691)

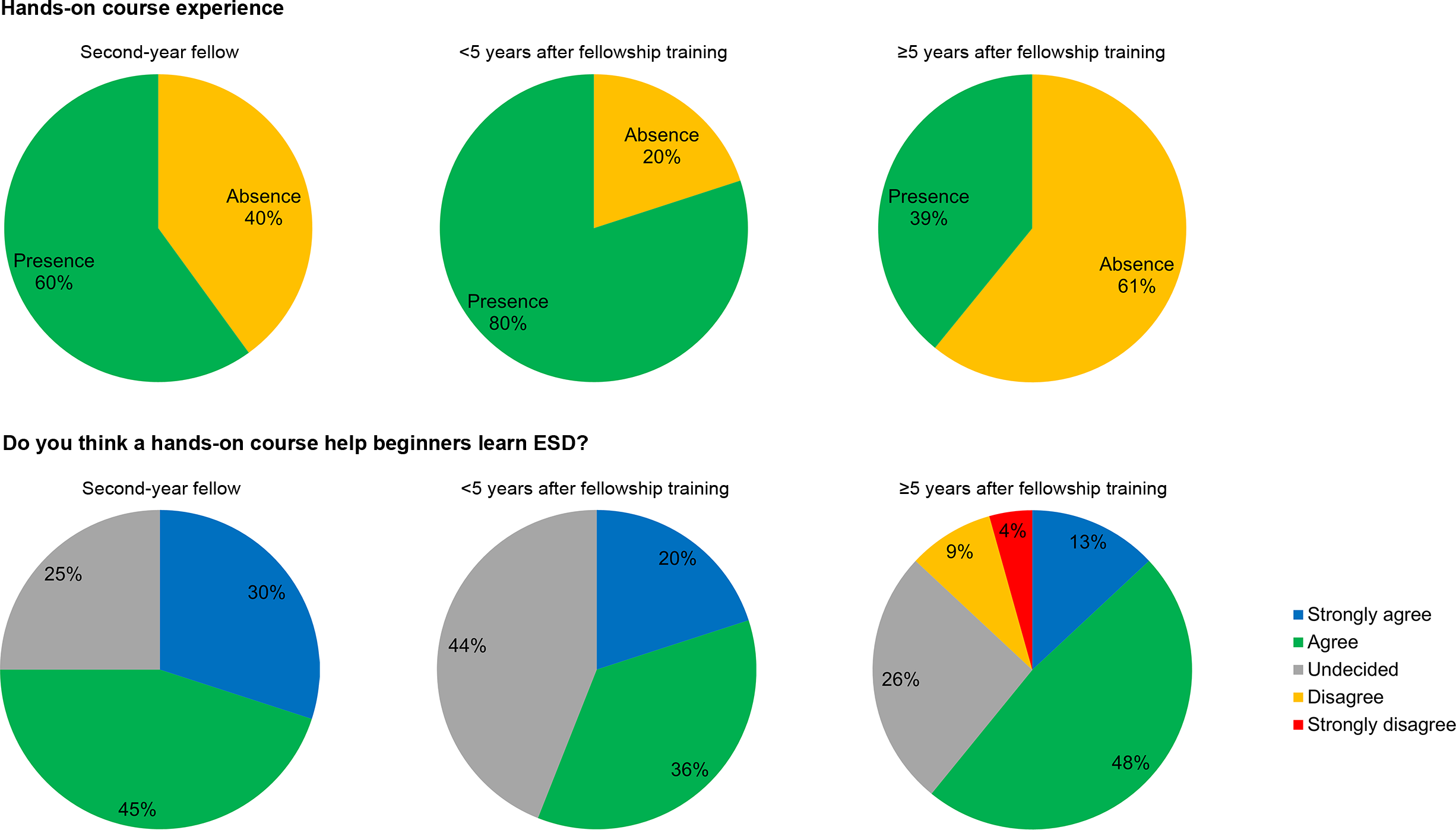

Supplement: S1 Fig — (A) Overall experience of a hands-on course during or after the training period. (B) Awareness of a hands-on course. The mean scores were 4.1, 3.8, and 3.6 in the second-year fellow, <5 years after fellowship training, and ≥5 years after fellowship training groups, respectively (P = 0.067). ESD, endoscopic submucosal dissection. (TIF) [file pone.0232691.s001.tif]
