# Supplementary material for: Current status and trend in training for endoscopic submucosal dissection: A nationwide survey in Korea
Source: PLoS One. 2020 May 8;15(5):e0232691. doi: 10.1371/journal.pone.0232691 (PMC7209322; doi:10.1371/journal.pone.0232691)
Supplement: S1 Table — (DOCX) [file pone.0232691.s002.docx]

| Table S1. Differential characteristics according to the current position | | | | | |
| --- | --- | --- | --- | --- | --- |
| Variable | | Second-year fellow | < 5 years after fellowship training | ≥ 5 years after fellowship training | P-value |
| Number, n | | 20 | 25 | 23 |  |
| Age, year, mean±SD | | 33.7±1.7 | 37.2±2.3 | 40.4±2.2 | <0.001 |
| Sex, n (%) | |  |  |  | 0.120 |
|  | Male | 10 (50.0) | 18 (72.0) | 18 (78.3) |  |
|  | Female | 10 (50.0) | 7 (28.0) | 5 (21.7) |  |
| Performing ESD independently | | 2 (10.0) | 24 (96.0) | 23 (100.0) | <0.001 |
| Major field of ESD, n (%)^a^ | |  |  |  |  |
|  | Esophagus | 0 (0.0) | 10 (40.0) | 9 (39.1) | 0.004 |
|  | Stomach | 14 (70.0) | 21 (84.0) | 22 (95.7) | 0.071 |
|  | Duodenum | 0 (0.0) | 1 (4.0) | 3 (13.0) | 0.170 |
|  | Colon | 3 (15.0) | 12 (48.0) | 11 (47.8) | 0.039 |
|  | Rectum | 5 (25.0) | 11 (44.0) | 14 (60.9) | 0.061 |
| ^a^Multiple responses were allowed. ESD, endoscopic submucosal dissection; SD, standard deviation | | | | | |
