# Supplementary material for: Current status and trend in training for endoscopic submucosal dissection: A nationwide survey in Korea
Source: PLoS One. 2020 May 8;15(5):e0232691. doi: 10.1371/journal.pone.0232691 (PMC7209322; doi:10.1371/journal.pone.0232691)
Supplement: S2 Table — (DOCX) [file pone.0232691.s003.docx]

| Table S2. Experience and demand of hands-on course | | | | | | | |
| --- | --- | --- | --- | --- | --- | --- | --- |
| Variable | | | Second-year fellow | < 5 years after fellowship training | ≥ 5 years after fellowship training | Total | *P*-value |
| Participants who experienced hands-on course | | |  |  |  |  |  |
|  | Number, n | | 12 | 20 | 9 | 41 |  |
|  | Type of hands-on model, n (%) | |  |  |  |  |  |
|  |  | Esophagus | 3 (25.0) | 6 (30.0) | 2 (22.2) | 11 (26.8) | >0.999 |
|  |  | Stomach | 11 (91.7) | 20 (100.0) | 9 (100.0) | 40 (97.5) | 0.512 |
|  |  | Duodenum | 0 (0.0) | 0 (0.0) | 1 (11.1) | 1 (2.4) | 0.220 |
|  |  | Colorectum | 1 (8.3) | 1 (5.0) | 0 (0.0) | 2 (4.9) | >0.999 |
|  | Hands-on course program, n (%)^a^ | |  |  |  |  |  |
|  |  | By hospital | 8 (66.7) | 11 (55.0) | 9 (100.0) | 28 (68.3) | 0.041 |
|  |  | By conference | 2 (16.7) | 5 (25.0) | 3 (33.3) | 10 (24.4) | 0.652 |
|  |  | By endoscopic device company | 5 (41.7) | 10 (50.0) | 1 (11.1) | 16 (39.0) | 0.118 |
| Participants who did not experience hands-on course | | |  |  |  |  |  |
|  | Number, n | | 8 | 5 | 14 | 27 |  |
|  | Desired hands-on model, n (%) | |  |  |  |  |  |
|  |  | Esophagus | 2 (25.0) | 3 (60.0) | 6 (42.9) | 11 (26.8) | 0.450 |
|  |  | Stomach | 6 (75.0) | 1 (20.0) | 3 (21.4) | 10 (24.4) | 0.040 |
|  |  | Duodenum | 1 (12.5) | 0 (0.0) | 5 (35.7) | 6 (14.6) | 0.259 |
|  |  | Colorectum | 6 (75.0) | 4 (80.0) | 7 (50.0) | 17 (41.5) | 0.369 |
| ^a^Multiple responses were allowed. | | | | | | | |
