# Supplementary material for: Current status and trend in training for endoscopic submucosal dissection: A nationwide survey in Korea
Source: PLoS One. 2020 May 8;15(5):e0232691. doi: 10.1371/journal.pone.0232691 (PMC7209322; doi:10.1371/journal.pone.0232691)
Supplement: S1 Appendix — (DOCX) [file pone.0232691.s004.docx]

**Appendix 1. Survey questionnaire**

1. Questions about sex, age, and training status

A. What is your sex?

a. Male

b. Female

B. How old are you?

C. Are you currently in fellowship training?

a. Under training (move to Question 1-1)

b. Training completed (move to Question 1-2)

1-1. Questions for participants currently under fellowship training

1. How many years has it been since you started fellowship training?
2. At which hospital are you receiving your fellowship training?
3. What is the major focus of your clinical training? (multiple responses permitted)
4. Upper gastrointestinal tract
5. Lower gastrointestinal tract
6. Pancreas or biliary tract
7. Liver
8. Functional gastrointestinal disorder
9. Other.
10. Do you currently perform ESD independently?
11. Yes
12. No
13. Other.
14. What is your primary target organ for ESD? (multiple responses permitted)
15. Esophagus
16. Stomach
17. Duodenum
18. Colon
19. Rectum
20. Etc.
21. How much time do you spend on ESD during your working hours?
22. Total clinical care-related time
23. ESD-related time

(move to Question 2)

1-2. Questions for participants who have completed their fellowship training

A. How many years did it take you to complete your fellowship training?

1. How many years have passed since you completed your fellowship training?
2. What is the major focus of your clinic? (multiple responses permitted)
3. Upper gastrointestinal tract
4. Lower gastrointestinal tract
5. Pancreas or biliary tract
6. Liver
7. Functional gastrointestinal disorder
8. Other.
9. Do you currently perform ESD independently?
10. Yes
11. No
12. Etc.
13. What is your primary target organ for ESD? (multiple responses permitted)
14. Esophagus
15. Stomach
16. Duodenum
17. Colon
18. Rectum
19. Other.
20. How much time do you spend on ESD during your working hours?
21. Total clinical care-related time
22. ESD-related time

(move to Question 2)

2. Questions regarding training methods

A. When did you start ESD observation?

1. During residency training
2. During the first-year fellowship
3. During the second-year fellowship
4. During the third-year (or higher) fellowship
5. After fellowship training
6. No with ESD observation
7. Other.

B. Did you have an additional role during observation? (multiple responses permitted)

1. No experience with ESD observation
2. No. I observed ESD cases without any other role.
3. Yes. I monitored the patient’s vital signs or sedation level.
4. Yes. I performed patient sedation for ESD.
5. Yes. I assisted with ESD procedures (*e.g.*, assistance with hypertonic saline injection or electrosurgical knife).
6. Other.

C. How many ESD cases did you observe before starting ESD procedures under supervision?

1. No experience with ESD observation
2. <10 cases
3. 10 – 50 cases
4. 50 – 100 cases
5. ≥100 cases
6. Other.

D. When did you start performing ESD procedures under supervision?

1. During residency training
2. During the first-year fellowship
3. During the second-year fellowship
4. During the third-year (or higher) fellowship
5. After fellowship training
6. No experience with ESD observation
7. Other.

E. How many ESD cases did you perform under supervision?

1. No experience with performing ESD under supervision
2. <10 cases
3. 10 – 20 cases
4. 20 – 30 cases
5. ≥30 cases
6. Other.

F. What was the target organ in your supervised ESD?

1. No experience with performing ESD under supervision
2. Esophagus
3. Upper third of the stomach
4. Middle third of the stomach
5. Lower third of the stomach
6. Duodenum
7. Colon
8. Rectum
9. Do you think that the training course in your hospital was systematic?
10. Strongly agree
11. Agree
12. Undecided
13. Disagree
14. Strongly disagree
15. Are you satisfied with your training course?
16. Strongly agree
17. Agree
18. Undecided
19. Disagree
20. Strongly disagree
21. What was the most wanted method for learning ESD?
22. Observation of ESD
23. Symposium or conference
24. Liver demonstration
25. Literature (*e.g.*, journal, book)
26. Video (*e.g.*, YouTube)
27. Hands-on course
28. ESD under supervision of an expert endoscopist
29. What were your preferred methods for learning ESD? (select up to three)
30. Observation of ESD
31. Symposium or conference
32. Liver demonstration
33. Literature (*e.g.*, journal, book)
34. Video (*e.g.*, YouTube)
35. Hands-on course
36. ESD under supervision of an expert endoscopist
37. Which method for learning ESD did you experience most often?
38. Observation of ESD
39. Symposium or conference
40. Liver demonstration
41. Literature (*e.g.*, journal, book)
42. Video (*e.g.*, YouTube)
43. Hands-on course
44. ESD under supervision of an expert endoscopist
45. What methods did you experience to learn ESD? (multiple responses permitted)
46. Observation of ESD
47. Symposium or conference
48. Liver demonstration
49. Literature (*e.g.*, journal, book)
50. Video (*e.g.*, YouTube)
51. Hands-on course
52. ESD under supervision of an expert endoscopist
53. Did you have experience with a hands-on ESD course using *in vivo* or *ex vivo* animal models?
54. Yes (move to Question 3-2)
55. No (move to Question 3-1)

3. Questions about hands-on ESD courses

3-1. Questions for participants with no experience with ESD hands-on courses

A. Do you think a hands-on course would help beginners to learn ESD?

1. Strongly agree
2. Agree
3. Undecided
4. Disagree
5. Strongly disagree

B. Why did you choose the above?

C. What ESD hands-on model would you like to experience in the future? (multiple responses permitted)

1. Esophagus
2. Stomach
3. Duodenum
4. Colorectum

(move to Question 4)

3-2. Questions for participants with experience with ESD hands-on courses

A. What ESD hands-on model did you experience? (multiple responses permitted)

1. Esophagus
2. Stomach
3. Duodenum
4. Colorectum

B. How did you experience the ESD hands-on model? (multiple responses permitted)

1. In the training hospital’s program
2. At a domestic medical conference
3. At an international medical conference
4. At a program organized by an endoscopic device company

C. Do you think a hands-on course helped you learn ESD?

1. Strongly agree
2. Agree
3. Undecided
4. Disagree
5. Strongly disagree

D. Why did you choose the above?

E. Do you think a hands-on course helps beginners to learn ESD?

1. Strongly agree
2. Agree
3. Undecided
4. Disagree
5. Strongly disagree

F. Why did you choose the above?

(move to Question 4)

4. Questions about general opinions

A. How many cases of ESD observation do you think are necessary before performing ESD under supervision?

B. How many cases of ESD under supervision do you think are necessary before performing ESD independently?

C. Please describe the training methods for ESD in your training hospital.

D. Please comment on important points to consider when starting ESD.
